# Phylogenetical Position versus Pollination Syndromes: Floral Trichomes of Central American and Mexican *Pinguicula*

**DOI:** 10.3390/ijms24098423

**Published:** 2023-05-08

**Authors:** Krzysztof Lustofin, Piotr Świątek, Vitor F. O. Miranda, Bartosz J. Płachno

**Affiliations:** 1 Department of Plant Cytology and Embryology, Institute of Botany, Faculty of Biology, Jagiellonian University in Cracow, 9 Gronostajowa St., 30-387 Cracow, Poland; krzysztof.lustofin@doctoral.uj.edu.pl; 2 Doctoral School of Exact and Natural Sciences, Jagiellonian University, 30-348 Cracow, Poland; 3 Institute of Biology, Biotechnology and Environmental Protection, Faculty of Natural Sciences, University of Silesia in Katowice, 9 Bankowa St., 40-007 Katowice, Poland; 4 Laboratory of Plant Systematics, School of Agricultural and Veterinarian Sciences, São Paulo State University (Unesp), Jaboticabal 14884-900, Brazil

**Keywords:** *Pinguicula*, flower micromorphology, trichomes, phylogeny, carnivorous plants

## Abstract

Central American and Mexican *Pinguicula* species are characterized by enormous divergence in size and color of flowers and are pollinated by butterflies, flies, bees, and hummingbirds. It is known that floral trichomes are key characters in plant–pollinator interaction. The main aim of our study was to verify our hypothesis that the distribution and diversity of non-glandular and glandular trichomes are related to the pollinator syndromes rather than the phylogenetic relationships. The studied sample consisted of Central American and Mexican species. In our study, we relied on light microscopy and scanning electron microscopy with a phylogenetic perspective based on *ITS* DNA sequences. The flower morphology of species pollinated by butterflies and hummingbirds was similar in contrast to species pollinated by flies and bees. Species pollinated by butterflies and hummingbirds contained low diversity of non-glandular trichomes, which occurred mostly in the tube and basal part of the spur. Surprisingly, in *P. esseriana* and *P. mesophytica*, non-glandular trichomes also occurred at the base of lower lip petals. In the case of species pollinated by flies/bees, we observed a high variety of non-glandular trichomes, which occurred on the surface of corolla petals, in the tube, and at the entrance to the spur. Furthermore, we did not identify any non-glandular trichomes in the spur. The capitate glandular trichomes were of similar morphology in all examined species. There were minor differences in the shape of the trichome head, as well as the length and the number of stalk cells. The distribution and the diversity of non-glandular and glandular trichomes and pollinator syndromes were mapped onto a phylogenetic reconstruction of the genus. Most micromorphological characters appear to be associated more with floral adaptation to pollinators and less with phylogeny.

## 1. Introduction

### 1.1. Plant Trichomes and Their Function in General

Trichomes are epidermal appendages that can occur on the surfaces of both vegetative (leaves, stems, bracts, and roots) and generative organs (sepals, petals, stamens, gynoecium, seeds, and fruits) of plants. They are intermediate structures between papillae and emergencies and may be classified into two distinct groups: glandular and non-glandular trichomes [1]. Plant trichomes have various functions that are related to the types of organs on which they occur. In the case of vegetative organs, they may protect plants against environmental factors, as well as against various types of herbivores and pathogens, glandular trichomes secreting mucilage, lipophilic substances, and resins. In addition, they assist in getting rid of unnecessary substances (e.g., salts) or absorb water [1,2,3,4,5]. Moreover, trichomes may also participate in plant organ movement and constitute an element of the plant biomechanical system as an additional reservoir of hydrostatic pressure [6]. Trichomes may also attach plants to the surface, e.g., in aquatic rheophytic species [7,8]. Glandular trichomes are crucial in the case functionality of carnivorous plant traps [9], especially in members of Lentibulariaceae, Byblidaceae, some Droseraceae, and Plantaginaceae (*Philcoxia*). Some trichomes allure and help to catch prey (mucilage trichomes), while others produce digestive enzymes and absorb nutrients [10,11,12,13,14,15,16].

Werker [1] drew attention to a particular paradox, where the trichomes on the reproductive organs are supposed to produce repellent substances (against undesirable animals). On the other hand, the plant should attract pollinators. Thus, plants, using various glandular trichomes which produce volatile organic compounds, have a complicated dialogue with animals [17,18,19]. In this unique game, nectar also plays a significant role [20,21,22]. Nectar can be secreted by trichomes, the most common epidermal nectaries [23]. There are three main types of nectary trichomes: unicellular trichomes, multicellular linear trichomes, and multicellular capitate trichomes [24]. 

### 1.2. Plant Trichomes and Glands in Carnivorous Plants

Carnivorous plants are not monophyletic, because carnivorous syndromes have evolved independently in about 10 lineages of flowering plants [25,26,27,28]. For this reason, there is a great diversity of traps and trap glandular structures among carnivorous plants [9,29,30,31,32]. In carnivorous plant traps, the glandular apparatus may be organized as glandular hairs (trichomes, which are of epidermal origin), emergences (tentacles, which contain vascular tissue elements), or glands sunken in other tissues or the glandular epidermis. The organization of these structures varies enormously, from trichomes with few cells in carnivorous Lamiales (in *Byblis*, *Pinguicula*, *Genlisea*, and *Utricularia*), *Philcoxia*, and some Droseraceae (in *Aldrovanda* and *Dionaea*) to complex mucilage emergences in carnivorous Nepenthales or giant glands in *Nepenthes* [9,10,13,31,33,34,35,36,37]. In carnivorous plant traps, there are various glandular systems: trichomes/glands for attracting prey, which are primarily responsible for the production of nectar and olfactory attractants; glands for trapping prey that produce mucilage, viscoelastic liquid, or resin; digestive and absorptive glands [31,32]; trichomes/glands at the outer surface of traps. The last group is also diverse in terms of structure and function [38,39,40]. However, some trap glands have multiple roles, e.g., glandular emergences in *Drosera*, which produce both mucilage for trapping prey and digestive enzymes, as well as absorb nutrients [41,42]. According to Heslop-Harrison [35] and Juniper et al. [9], regardless of digestive–absorptive trichome/gland structure and origin, all include a terminal element (glandular cells that secrete digestive enzymes and absorb nutrients from dissolved prey bodies), a barrier element (an endodermoid cell or cells with lateral cell walls similar to a Casparian strip), and a basal cell or cells (a reservoir element) that connect the gland with other tissue cells.

### 1.3. State of the Art of Trichomes in Lentibulariaceae, Especially in the Genus Pinguicula

In Lentibulariaceae (genera: *Utricularia*, *Genlisea*, and *Pinguicula*), the spur is treated as a nectary, and nectar is produced by glandular capitate trichomes [43,44,45,46]. However, some corolla glandular trichomes may produce volatile compounds [47,48]. It should be noted that our knowledge regarding the function of glandular structures, located on the corolla and surface of generative organs, is far from being well understood. There is still a lack of experimental proof presenting various kinds of chemical compounds produced and secreted by particular types of trichome. In addition, it appears important to study the ultrastructure of these trichomes during various stages of flower development. We also do not know whether the ultrastructure of glands is similar between *Pinguicula* species that differ in terms of the type of pollinator (only a few species pollinated by butterflies were examined using a transmission electron microscope [46]). 

In addition to glandular trichomes in *Pinguicula* flowers, numerous non-glandular clavate trichomes (multicellular, uniseriate non-glandular trichomes, with clavate-shaped morphology) occur at the corolla. These trichomes had important value in the taxonomy of *Pinguicula*. Traditional infrageneric classification of *Pinguicula* was proposed on the basis of their morphological diversity [49]. Lustofin and coauthors [50] showed that non-glandular trichomes play the role of edible trichomes in some *Pinguicula* species, mainly classified as bee-pollinated species originating from Central America. However, for *Pinguicula* species that are pollinated by other pollinator groups (Lepidoptera and hummingbirds), trichomes in the flowers play a role other than that of a floral reward. Furthermore, it is known that floral trichomes are the key character in plant–pollinator interaction. Consequently, we aimed to verify the following two hypotheses: that both the distribution and diversity of non-glandular and glandular trichomes are connected to a type of pollinator, and that the distribution and diversity of non-glandular and glandular trichomes are more closely connected with the phylogenetical position.

## 2. Results

### 2.1. Flowers Micromorphology Analysis

#### 2.1.1. Species with Psychophily Syndrome (a Set of Features Found in Butterfly-Pollinated Flowers)

**Figure 1:**
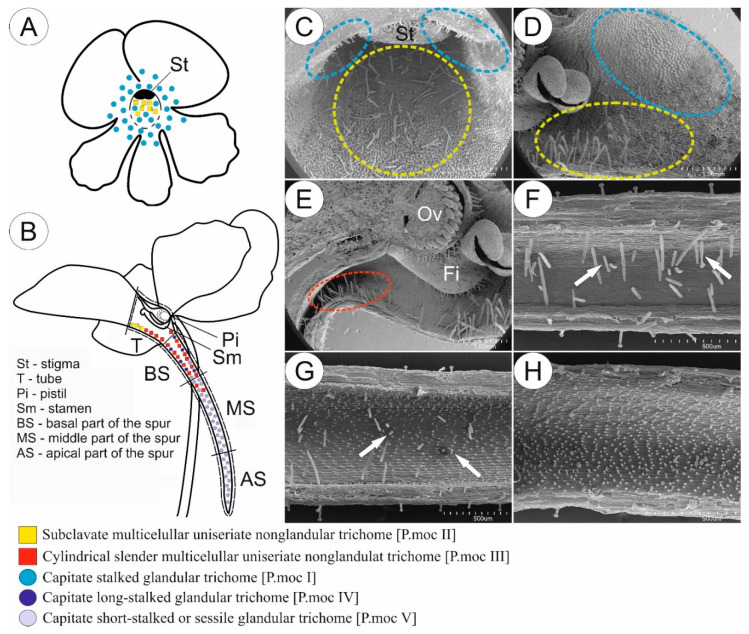
Sketch (**A**,**B**) and morphology (**C**–**H**) of *P. moctezumae* flower as a representative of a group of species pollinated by butterflies. (**A**,**B**) Front (**A**) and longitudinal section (**B**) of flower showing the distribution of various glandular and non-glandular trichomes. (**C**,**D**) Entrance to the tube; note the presence of subclavate multicellular non-glandular trichomes in front of the tube (yellow circle) and glandular trichomes located at the base of corolla petal around the entrance to the tube (blue circle); scale bars = 1 mm. (**E**) Tube with generative organs in front and numerous cylindrical multicellular non-glandular trichomes (red circle) at boundary with basal part of the spur; note the presence of glandular trichomes on the outer surface of ovary (Ov) and filament (Fi); scale bar = 1 mm. (**F**) Basal part of the spur; note the lack of papillae, numerous cylindrical non-glandular trichomes, and rare long-stalked glandular trichomes (arrow); scale bar = 500 µm. (**G**) Boundary between basal and middle part of the spur; note the presence of papillae and short-stalked glandular trichomes (arrow); scale bar = 500 µm. (**H**) Middle part of the spur; only short-stalked glandular trichomes are present, whereas non-glandular and long-stalked glandular trichomes are absent; scale bar = 500 µm.

**Figure 2:**
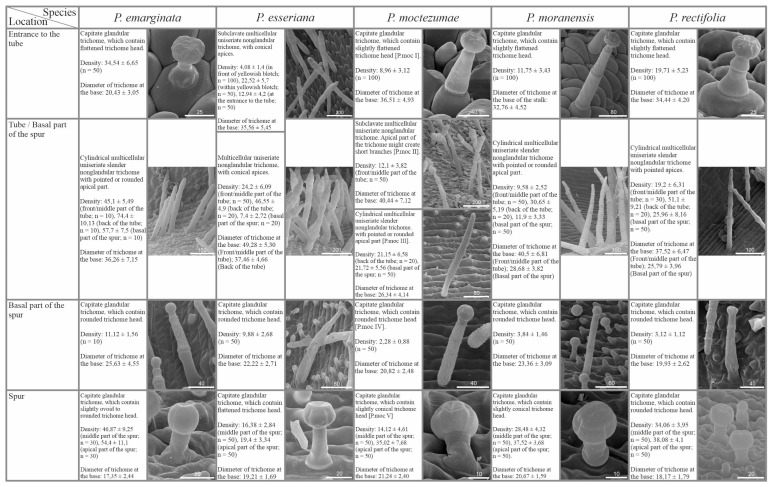
Micromorphological description including diameter at the base (mean in µm ± SD; n = 50) and density (number of trichomes/mm^2^) of various types of glandular and non-glandular trichomes, occurring in a particular part of examined *Pinguicula* flowers pollinated by butterflies (scale bars represent the length in µm).

In most of the examined species pollinated by butterflies, we observed glandular trichomes of different stalk lengths (in *P. emarginata*, only short-stalked glandular trichomes were present) and varying densities depending on the species (Figure 1A,C,D and Figure 2). Glandular trichomes occurred at the base of the petals of corolla around the entrance to the tube. The exception was *P. esseriana*, in which subclavate non-glandular trichomes were distributed at the base of the middle petal of the lower lip, within and around a greenish-yellowish blotch. The density of these trichomes varied. The highest density of trichomes was observed within the greenish-yellowish blotch, while the density around the greenish-yellowish blotch was lower (Figure 2). In few instances, in *P. esseriana*, we identified single short-stalked capitate glandular trichomes at the entrance to the tube. In species with psychophily syndrome, the middle part and edges of petals were without any trichomes (Figure 1A).

The generative organs were located at the front of the tube. The outer surface of the ovary wall was densely covered by capitate long-stalked glandular trichomes (Figure 1B,E and Figure 2) in all examined species. Cylindrical non-glandular trichomes were located at the front and middle part of the tube below generative organs, except for *P. moctezumae* and *P. esseriana*. In *P. moctezumae*, subclavate non-glandular trichomes (Figure 1B–D and Figure 2) were observed at the entrance and in front of the tube, whereas, in *P. esseriana*, subclavate non-glandular trichomes were distributed within the entire tube, including at the back of the tube. The density of non-glandular trichomes at the front and middle part of the tube varied depending on the species (Figure 2). At the back of the tube, on the border with the basal part of the spur, numerous non-glandular trichomes of the same morphological type as at the front of the tube were observed, except *P. moctezumae*, which contained the second morphological type of cylindrical non-glandular trichomes in the tube (Figure 1B,E and Figure 2). The density of non-glandular trichomes at the back of the tube was higher than that in the front and middle parts of the tube (Figure 2). In *P. esseriana,* papillae occurred at the back of the tube, whereas they were absent in other studied species pollinated by butterflies.

*P. emarginata* and *P. esseriana* had a strongly reduced basal part of the spur compared with other examined species. Furthermore, in these species, papillae occurred in the basal part of the spur, whereas, in *P. moctezumae*, *P. moranensis*, and *P. rectifolia*, papillae were absent. However, in the examined species pollinated by butterflies, cylindrical non-glandular trichomes and capitate long-stalked glandular trichomes occurred in the basal part of the spur (Figure 1B,F and Figure 2). The density of both trichome types varied, depending on the species (Figure 2). In all examined species, only capitate short-stalked glandular trichomes (nectary trichomes) of different densities, depending on the species (Figure 1B,H and Figure 2), were observed. They were located in the middle and apical part of the spur. However, in all examined species, the density of nectary trichomes, located in the spur, increased toward the apical part (Figure 2). Single cylindrical non-glandular trichomes occurred at the boundary between the basal and middle parts of the spur (Figure 1B,G and Figure 2). In all studied species, papillae were present in the middle and apical parts of spur.

#### 2.1.2. Species with Myophily/Mellitophily Syndrome (a Set of Features Found in Fly/Bee-Pollinated Flowers)

**Figure 3:**
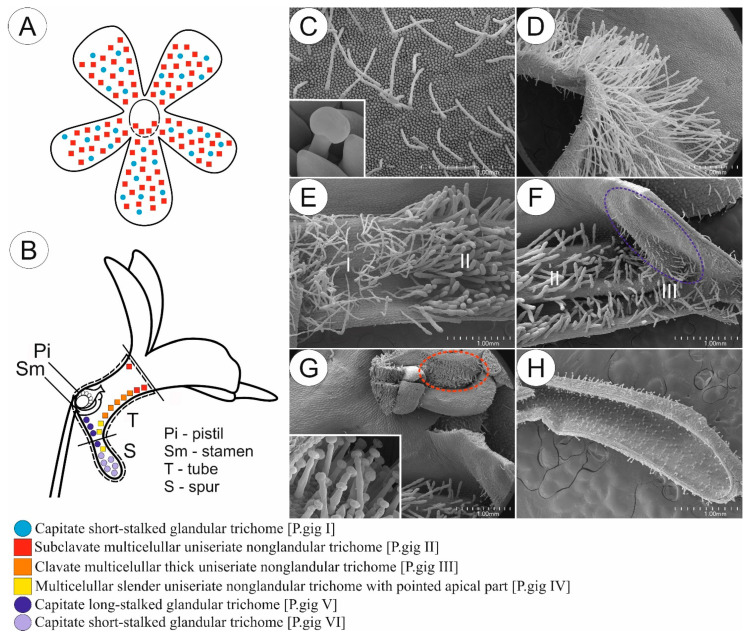
Sketch (**A**,**B**) and morphology (**C**–**H**) of *P. gigantea* flower as a representative of a group of species pollinated by flies and/or bees. (**A**,**B**) Front (**A**) and longitudinal section (**B**) of flower showing the distribution of various glandular and non-glandular trichomes. (**C**) Petal of corolla with evenly distributed subclavate elongated slender non-glandular trichomes and short-stalked glandular trichomes (insert); scale bar = 1 mm. (**D**) Entrance to the tube; note the abundance of non-glandular trichomes; scale bar = 1 mm. (**E**) Front of the tube with boundary between distribution of subclavate elongated slender (I) and subclavate thick (II) non-glandular trichomes; scale bar = 1 mm. (**F**) Back of the tube; note the presence of long-stalked glandular trichomes and papillae located above entrance to the spur (blue circle) and multicellular uniseriate non-glandular trichomes with pointed apical part at the entrance to the spur (III); scale bar = 1 mm. (**G**) Generative organs located at the back of tube; note the presence of glandular trichomes (insert) on the outer surface of the ovary (red circle); scale bar = 1 mm. (**H**) Spur with evenly distributed short-stalked glandular trichomes; note the lack of papillae inside of the spur; scale bar = 1 mm.

**Figure 4:**
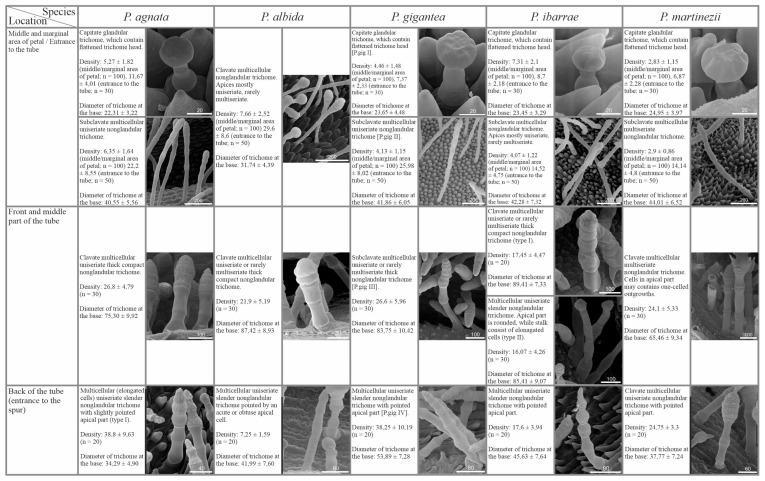

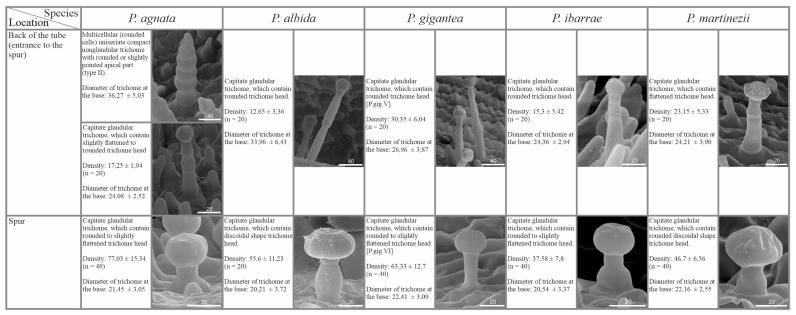
Micromorphological description including diameter at the base (mean in µm ± SD; n = 50) and density (number of trichomes/mm^2^) of various types of glandular and non-glandular trichomes, occurring in a particular part of examined *Pinguicula* flowers pollinated by flies and/or bees (scale bars represent the length in µm).

In most of the examined species pollinated by flies and/or bees, subclavate non-glandular trichomes and short-stalked capitate glandular trichomes occurred within the petals of the corolla. The density of both glandular and non-glandular trichomes was lower at the edges and middle part of the petals, while it was higher at the entrance to the tube (Figure 3A,C,D and Figure 4). The exception was *P. albida*, in which clavate non-glandular trichomes occurred only in the middle part and at the base of corolla petals. Similarly to other species pollinated by flies and/or bees, the density of non-glandular trichomes in *P. albida* was lower at the middle part of the petals than at the entrance to the tube (Figure 4).

Within the tube, 2–3 morphological types of non-glandular trichomes were observed in examined species. The front and middle parts of the tube bottom surface were mainly covered with one morphological type of non-glandular trichomes, depending on the species from subclavate to clavate, with comparable density (Figure 3B,E,F and Figure 4). The exception was *P. ibarrae*, which contained two morphological types of non-glandular trichomes in the front and middle part of the tube: clavate, thicker and more compact (type I), and slender with more elongated cells and rounded apices (type II). Their density was lower than in the case of the remaining species (Figure 4). The generative organs were located deep at the back of the tube, above the entrance to the spur. The outer surface of the ovary wall was densely covered by capitate glandular trichomes in all examined species (Figure 3G). Most of the examined species contained one morphological type of non-glandular trichome, located below the generative organs at the entrance to the spur. Non-glandular trichomes varied in terms of shape, from slender with elongated cylinder concave-shaped cells and a pointed apical part to clavate, depending on the species. However, in the case of *P. agnata*, two morphological types of non-glandular trichomes were observed at the back of the tube: slender with elongated cylinder concave shaped cells (type I), and thicker with more rounded compact cells (type II). The density of these trichomes varied, depending on the species (Figure 4). Additionally, in all examined species, papillae and long-stalked capitate glandular trichomes were identified on the back wall of the tube below the generative organs. The density of glandular trichomes varied between examined species (Figure 3B,F and Figure 4).

Capitate short-stalked glandular trichomes (nectary trichomes) were evenly distributed within the spur. These glandular trichomes had different densities depending on the species (Figure 3B,H and Figure 4). In *P. agnata*, *P. ibarrae*, and *P. martinezii*, papillae were present throughout the spur, whereas, in *P. albida* and *P. gigantea*, the occurrence of papillae was limited only to the entrance to the spur.

#### 2.1.3. Species with Ornithophily Syndrome (a Set of Features Found in Bird-Pollinated Flowers)

**Figure 5:**
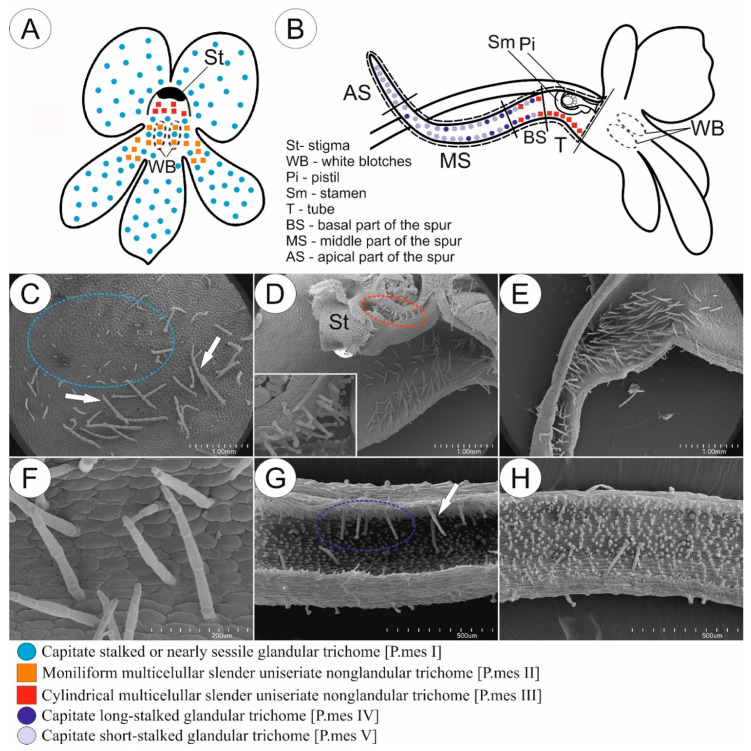
Sketch (**A**,**B**) and morphology (**C**–**H**) of *P. mesophytica* flower as a representative of a group of species pollinated most probably by hummingbirds. (**A**,**B**) Front (**A**) and longitudinal section (**B**) of flower showing the distribution of various glandular and non-glandular trichomes. (**C**) Entrance to the tube (including white blotches region); note the presence of moniliform multicellular uniseriate slender non-glandular trichomes (arrow) and stalked glandular trichomes (blue circle); scale bar = 1 mm. (**D**) Front of the tube with present generative organs; note the presence of glandular trichomes (insert) on the outer surface of the ovary (red circle); scale bar = 1 mm. (**E**,**F**) Back of the tube; note the presence of cylindrical multicellular uniseriate slender non-glandular trichomes (**F**), which are prolific at the entrance to the spur; scale bars = 1 mm and 200 µm. (**G**) Boundary between basal and middle part of the spur; note the presence of papillae, cylindrical non-glandular trichomes (arrow), and long-stalked glandular trichomes (blue circle); scale bar = 500 µm. (**H**) Middle part of the spur with visible short-stalked and long-stalked glandular trichomes; scale bar = 500 µm.

**Figure 6:**
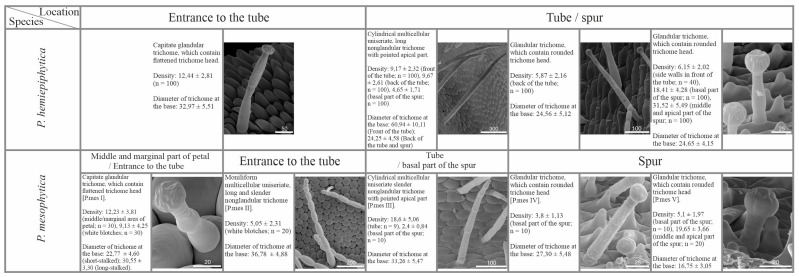
Micromorphological description including diameter at the base (mean in µm ± SD; n = 50) and density (number of trichomes/mm^2^) of various types of glandular and non-glandular trichomes, occurring in a particular part of examined *Pinguicula* flowers pollinated most probably by hummingbirds (scale bars represent the length in µm).

In *P. mesophytica,* capitate glandular trichomes were distributed on the entire surface of the corolla. The density of these trichomes varied. It was higher at the edges and in the middle part of the petals, but lower in the area where petals of the lower lip fused together, including white blotches (at the entrance to the tube). Additionally, moniliformis non-glandular trichomes were present at the lower lip within and around white blotches (Figure 5A,C and Figure 6). In the case of *P. hemiepiphytica,* similarly to species pollinated by butterflies, capitate glandular trichomes of different stalk length occurred within the corolla. The distribution of capitate glandular trichomes was limited to the base of corolla petals around the entrance to the tube (Figure 6).

The generative organs were located at the front of the tube. In ornithogamous species, the outer surface of the ovary wall was densely covered with capitate glandular trichomes (Figure 5D). In *P. hemiepiphytica*, the tube was elongated. Cylindrical non-glandular trichomes dominated the surface of the bottom wall of the tube. These trichomes were very long at the front of the tube and shorter at the back of the tube. The density of non-glandular trichomes was comparable in the front and back of the tube (Figure 6). Additionally, short-stalked capitate glandular trichomes occurred on the side walls at the front of the tube. Their morphology was similar to trichomes present in the spur, except for density, which was significantly lower in the former case (Figure 6). Capitate glandular trichomes present at the back of the tube had a very long stalk and narrow trichome head. They were less frequent than cylindrical non-glandular trichomes (Figure 6). In *P. mesophytica*, the tube was shorter and contained only cylindrical non-glandular trichomes, whose occurrence was prolific (Figure 5B,D–F and Figure 6).

In *P. hemiepiphytica* and *P. mesophytica*, the spur was dominated by capitate short-stalked glandular trichomes (nectary trichomes). Their density within the spur increased toward the apical part (Figure 5B,G,H and Figure 6). Additionally, in the *P. hemiepiphytica* spur, single cylindrical non-glandular trichomes and capitate long-stalked glandular trichomes occurred. In the case of *P. mesophytica*, cylindrical non-glandular trichomes occurred at the basal part of the spur. Their density at the basal part of the spur was significantly lower than in the tube (Figure 5B,G and Figure 6). The capitate long-stalked glandular trichomes were observed only at the basal part of the spur, and their density was low (Figure 6). In both examined species, papillae were present in the spur.

### 2.2. Phylogenetic Analysis

**Figure 7:**
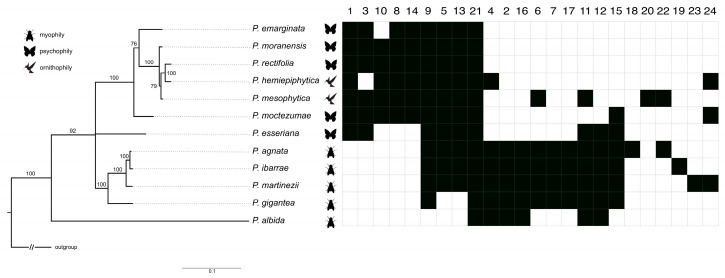
Bayesian tree based on ITS region (rDNA) for *Pinguicula* (Lentibulariaceae) with a distribution of flower morphological characters (for a description of characters, see Table 1). When more than one sequence was applied for the same species, the branch was collapsed. The morphological characters were clustered on the basis of Euclidean distances with Morpheus (https://software.broadinstitute.org/morpheus/; accessed on 6 May 2022). Numbers above the branches are posterior probabilities.

The tree based on the ITS region (rDNA) dataset revealed that myophily pollination syndrome is a plesiomorphic condition. Moreover, the topology suggested that psychophily is a syndrome derived from myophilous pollination, while ornithophily seems to be derived from psychophily (Figure 7).

The phylogenetic tree revealed two major and well-supported (posterior probabilities = 100%) monophyletic groups, represented by distinct pollination syndromes: *P. agnata*–*P. ibarrae*–*P. martinezii*–*P. gigantea* (myophily; *P. agnata*–*P. gigantea* clade) and *P. emarginata*–*P. moranensis*–*P. rectifolia*–*P. hemiepiphytica*–*P. mesophytica*–*P. moctezumae* (psychophily and ornithophily; *P. emarginata–P. moctezumae* clade). *P. esseriana* (psychophily) and *P. albida* (myophily) were not nested in any of the referred clades (Figure 7).

### 2.3. Tracing Analysis of Flower Morphological Characters 

The character tracing analysis revealed some morphological traits of the flowers as synapomorphies, supporting the monophyly of particular clades. The *P. agnata–P. ibarrae–P. martinezii–P. gigantea* clade had the following synapomorphies: non-glandular trichomes on the middle part and edges of corolla petals (No. 7; see Figure 7 and Table 1) and non-glandular trichomes, with elongated cylinder concave shaped cells (No. 17; see Figure 7 and Table 1). The *P. emarginata–P. moctezumae* clade had synapomorphies such as short-stalked capitate glandular trichomes at the base of corolla petals of upper lip (No. 8; see Figure 7 and Table 1) and apical to obtuse pointed, slender non-glandular trichomes (No. 14; see Figure 7 and Table 1).

Species with myophily pollination syndrome were characterized by the presence of clavate non-glandular trichomes (No. 16; see Figure 7 and Table 1), a shortened spur (No. 2; see Figure 7 and Table 1), and an elongated tube (No. 4; see Figure 7 and Table 1), while species with psychophily/ornithophily pollination syndromes had an elongated spur (No. 1; see Figure 7 and Table 1) and, in general, a short tube (No. 3; see Figure 7 and Table 1); the exception was *P. hemiepiphytica*, in which the tube is elongated. The glandular trichomes on the middle part and edges of corolla petals (No. 6; see Figure 7 and Table 1) occurred within the *P. agnata*–*P. gigantea* clade; however, this feature was also derived independently for *P. mesophytica*. The presence of long-stalked capitate glandular trichomes at the base of corolla petals (No. 10; see Figure 7 and Table 1) occurred in the *P. emarginata–P. moctezumae* clade, except *P. emarginata*, which lacked these trichomes. The presence of papillae at the entrance to the spur (No. 12; see Figure 7 and Table 1) occurred in the *P. agnata*–*P. gigantea* clade, as well as in *P. esseriana* and *P. albida*. Furthermore, the presence of subclavate non-glandular trichomes (No. 15; see Figure 7 and Table 1) occurred in the *P. agnata*–*P. ibarrae*–*P. martinezii*–*P. gigantea* clade, as well as *P. esseriana* and *P. moctezumae*. Similar non-glandular trichomes on the corolla petals of the lower lip occurred (No. 11; see Figure 7 and Table 1) in *P. agnata*, *P. albida*, *P. esseriana*, *P. gigantea*, *P. ibarrae*, and *P. martinezii*, as well as evolved in *P. mesophytica*. Morphological features of non-glandular trichomes such as a smooth cuticle surface (No. 22; see Figure 7 and Table 1) and few celled branches (No. 24; see Figure 7 and Table 1) occurred in far-related species (Figure 7).

The character tracing analysis showed that the remaining morphological features of flowers, such as the presence of non-glandular trichomes, with compact rounded cells, moniliform non-glandular trichomes, non-glandular trichomes with rounded apical “head” cell, or non-glandular trichomes with outgrowths, were autapomorphies derived for *P. agnata, P. mesophytica, P. ibarrae*, and *P. martinezii*, respectively (Figure 7).

## 3. Discussion

### 3.1. Diversification and Possible Function of Glandular and Non-Glandular Trichomes of Central American Pinguicula and Mexican Species

This study aimed to determine whether floral traits of Central American and Mexican *Pinguicula* species are more a result of adaptation to particular groups of pollinators (pollination syndromes) or more closely related to phylogenetic relationships. To address our aims, we performed a detailed micromorphological analysis of floral traits with emphasis on the morphology, distribution, and density of various glandular and non-glandular trichomes. Subsequently, we reconstructed the phylogeny of examined *Pinguicula* species on the basis of ITS region (nrDNA) sequences and mapped selected micromorphological flower traits onto a tree to check their phylogenetic histories.

Most Central American and Mexican *Pinguicula* species are closely related according to phylogeny [51]. These species varied significantly in terms of sizes, shapes, and colors of flowers, which attract different types of pollinators: flies/bees (myophily/mellitophily syndromes) [52], butterflies (psychophily syndrome) [53,54], and likely hummingbirds (ornithophily syndrome) [55,56]. Flowers pollinated by butterflies and hummingbirds had similar morphology, characterized by a long spur, bilobed zygomorphic corolla with pink, violet, to red petals and the presence of bright contrasting signals in front of the flower entrance, whereas flowers pollinated by flies/bees had a different morphology characterized by a short spur and a nearly radial corolla with bright colors and without prominent contrasting color signals at the base of lower lip [49,55,57,58]. More recently, Zamudio et al. [59] described a new Central American *Pinguicula* species *P. warijia*, characterized by large, colored flowers and an elongated spur. This species is probably pollinated by butterflies, as the visitation of a two-tailed swallowtail *Papilio multicaudata* W.F.Kirby was observed and documented [59]. However, we think that a future study should be performed in order to prove that *Papilio multicaudata* is not only a flower guest, but also a pollinator.

SEM analysis showed that the micromorphology of glandular trichomes was conserved among examined *Pinguicula* species in which capitate glandular trichomes occurred. The only difference between glandular trichomes was the length of the stalk and the number of cells it consisted of (Figure 2, Figure 4 and Figure 6). In contrast, non-glandular trichomes showed considerable diversity, and their micromorphology and distribution seemed to be associated with pollination syndromes. The highest variation of non-glandular trichome morphology was revealed in species pollinated by flies/bees (3–4 types per species; Figure 4), and they were distributed throughout the flower (including most of the corolla, except *P. albida*), except for the short spur (Figure 3). In contrast, species pollinated by butterflies and hummingbirds were less diverse in the context of non-glandular trichome micromorphology (1–2 types per species), and the distribution of trichomes was restricted to the flower entrance, tube, and basal spur, with the highest density in front of the spur (Figure 1, Figure 2, Figure 5 and Figure 6). In a previous study, we revealed that, in the case of Central American species pollinated by flies and/or bees, these trichomes are edible and could possibly be eaten by pollinators [50]. However, trichomes of psychophily and ornithophily species seem to play a different role, as we did not observe any food material inside of them [50]. It is worth remembering that the occurrence of such edible tissue with various food reservoirs that attract pollinators has also been found in other plants. For instance, in orchids, we can distinguish the presence of edible pseudo-pollen created by the detachment or fragmentation of moniliformis trichomes in *Maxillaria* and *Polystachya* [60,61] or by the separation of a mature head with edible component cells from trichomes in *Dendrobium* [62], as well as the presence of edible trichomes on the labellum in *Polystachya*, *Grobya*, *Cyanaeorchis*, and *Vanilla* [60,63,64]. In general, *Pinguicula* non-glandular trichomes contain microcuticular striations on their surface (Figure 2, Figure 4 and Figure 6), which are prominent in psychophily and ornithophily species. These striations, under UV light, induce bright autofluorescence. In recent years, various researchers have shown that the butterfly *Papillio xuthus* Linnaeus [65,66] and many species of birds, including hummingbirds [67,68] have tetrachromatic vision, which enables them to perceive non-spectral UV light. The perception of UV plays an essential role in the context of intra- and intersexual signaling, as well as foraging. Accordingly, we suggest that non-glandular trichomes for psychophily and ornithophily species likely play a role as nectar guides and facilitate pollinators to find a way to the nectary within the spur. However, the presence of such trichomes in some plants might also be related to the natural selection of flower visitors (plant adaptation to deter or mechanically preclude insects from penetrating flowers and plundering the nectar). Such adaptations have been described, for example, in *Menyanthes trifoliata* (Menyanthaceae), where high-density trichomes, which play a role in the deterrence of nectar-thriving ants (*Lasius japonicus* Santschi), occur on petals [69]. In *U. multifida* and *U. tenella*, non-glandular trichomes, which densely cover the entrance to the tube and spur, may also constitute a mechanical barrier for non-pollinating visitors to prevent nectar theft [44]. Moreover, Fleischmann [70] suggested that in species such as *P. caerulea, P. ionantha, P. lutea, P. primuliflora, P. pumila* (from southeastern USA), and *P. debbertiana* (from Mexico), yellowish, whitish, or greenish trichomes occur near the corolla entrance, as mimicry of pollen. Admittedly, *Pinguicula* produce nectar in the spur; thus, a flower reward for pollinators is present. Nevertheless, even if such trichomes mimic pollen, they should be considered as an additional feature to lure pollinators.

In our previous work, we examined and described the structure of nectary trichomes, which produce and secrete nectar inside of the spur [46]. However, the function of other types of glandular trichomes with various locations remains unknown. However, we would like to propose some suggestions. In all examined *Pinguicula* species, regardless of the pollination syndrome, glandular trichomes occurred on corolla petals. Furthermore, in psychophily and ornithophily species, glandular trichomes were centered around the entrance to the flower (except *P. mesophytica*, in which trichomes occurred on the whole corolla), whereas, in the case of myophily/mellitophily species, they were more widely distributed on petals. Similarly to *Pinguicula*, glandular trichomes also occur on the palate of related genera *Utricularia* and *Genlisea*. For example, glandular trichomes were reported in *U. cornigera*, *U. nelumbifolia*, *U. dunlopii*, and *G. hispidula* [48,71,72]. According to the authors, the palate in the case of these species probably plays the role of an osmophores, attracting pollinators through the production and secretion of various scent compounds. We cannot exclude a similar olfactory function for corolla glandular trichomes of *Pinguicula* species. However, to test this hypothesis, histochemical studies, preferably supported by chemical composition analysis using chromatographic distribution, would be required.

### 3.2. Floral Adaptions to Particular Groups of Pollinators: Pollination Syndrome Concept

Flower morphology is highly influenced by selective pollinator pressure. *Pinguicula* species with psychophily/ornithophily syndrome have long spurs, as well as a short, dorsally oriented tube located inside generative organs, and prolific distributed trichomes on the bottom of the tube, which together impede or disable the penetration of the spur by animals other than pollinators. In contrast, species with myophily/mellitophily syndromes have a short spur, as well as wide and long tube with generative organs located at the end of the tube above the entrance to spur, which allows flower penetration. Due to these morphological flower adaptations, pollinators that differ in terms of mouth length (long- and short-tongued pollinators) can easily reach the reward (nectar) stored in the spur, simultaneously allowing reproductive success by pollination. Similar patterns of floral morphological adaptions to pollinators have been found in other genera. Johnson et al. [73] examined the evolution of pollination syndromes of orchids from *Disa* genus, among which different pollinators have been found. The authors showed that newly derived specific adaptations of flower morphology might prove attractive to particular (new) groups of pollinators, thus persisting in the population as convergences. For example, red flowers attract butterflies, night-scented flowers attract moths, long-spurred flowers attract long-tongued flies, the lack of spur in flowers attracts bees or wasps, and deceptive flowers attract carpenter bees. More recently published studies [74,75] revealed the appearance of some new floral characters within the *Fritillaria* genus related to floral morphology and reward, which led to shifts of principal pollinators. For instance, newly derived red and orange flowers in *F. recurve*, *F. gentneri*, and *F. imperialis*, as well as the alteration in their sugar and amino-acid content in nectar, have led to a pollinator shift for these species from insects to birds. Another interesting example of floral adaptation in the context of floral reward to particular pollinators provides us with two deceptive subspecies of *Disa spathulata*, which differ in chemical composition of fragrance produced by the lip blade. Only 24 out of 57 scent compounds were common to both subspecies. *D. spathulata* subsp. *spathulata* produces a scent dominated by fatty acids, whereas *D. spathulata* subsp. *tripartita* has a scent dominated by terpenoids. Results highlighted the flower scent-based specialization for the two closely related subspecies, for which the species-specific chemical composition of scent attracted particular bees: *Tetraloniella brevikeraia* and *Tetraloniella junodi*, respectively [76].

On the other hand, it should be emphasized that many studies addressed the question of whether pollination syndromes occur in nature or not. Specifically, recent research related to the pollination ecology of *P. moranensis* [77] showed that flowers of *P. moranensis* were visited most frequently by bees from *Sphecodes* genus (Halictidae) (88.9% of cases, within Teacalco population). These results appear surprising, considering that the morphological features of *P. moranensis* flowers (e.g., large, zygomorphic, and long-spurred flowers) indicate psychophily pollination syndrome. However, the results of Perez-Alva et al. were divergent from those obtained by Villegas and Alcalá [54], revealing that the most frequent visitors of *P. moranensis* flowers were Lepidoptera spp. (86% of cases) from five families (Pieridae, Papilionidae, Hesperiidae, Nymphalidae, and Lycanidae). Despite these observations, there is still a lack of hard evidence (such as SEM analysis of pollen load on insect body and its transfer to stigma), which could prove that these bees and/or butterflies are effective pollinators. Currently, we can consider them as “potential” pollinators; thus, further ecological research is needed. Additionally, Roguz et al. [78] prepared an experiment with false-color pictures (bee vision) of flowers for ornithophilous species *F. recurve* and *F. imperialis*, which proved that their corolla colors and the flower rewards within were also visible to bees. These observations do not support the pollination syndrome theory, related to the morphological specialization of flowers to a particular group of pollinators. Research related to the testing of pollination syndromes was also performed on a bigger number of species belonging to different families. For instance, Ollerton et al. [79] performed a test of the pollination syndromes on a large group of 482 species, belonging to 27 families and different communities. Results revealed that the primary pollinator was successfully predicted for only one-third of examined species. Moreover, species from only three families, Fabaceae, Apocynaceae, and Asteraceae had the best predictive ability of pollination syndromes. The authors emphasized that floral phenotypes are not only influenced by the adaptation to a particular group of pollinators but also modulated by series of other factors such as antagonistic floral visitors, mixtures of pollinators of different functional types, and pleiotropic effects on other plant traits. Another author came to similar conclusions. In the case of the Solanaceae family [80], within which some genera reflect a high variety of pollination syndromes and floral architecture (e.g., *Iochroma* and *Nicotiana*), species with different flower morphology share pollinators. Further floral diversification of species does not correlate with pollinator shift. This indicates that other factors are responsible for flower diversification, such as habitat shifts. A more recent example is the research of Krakos and Austin [81], who tested pollination syndromes among *Oenothera* species. They mapped nine floral traits (color of anthesis; scent; flower shape, symmetry, orientation, and brightness; anthesis time; nectar presence and location) into morphospace for 54 species from the *Oenothera* genus, constituting a monophyletic group of species pollinated by various animals (fly, bat, carrion fly, bee, butterfly, bird, beetle, mammal, moth/hawkmoth, and wasp). The obtained results did not support the pollination syndrome concept. The only pollination syndrome which could be accurately predicted using floral traits within this genus was moth pollination syndrome.

Assigning a species to a specific pollination syndrome only on the basis of its floral characteristics is not always a suitable approach. There are studies which showed that a given plant species is pollinated by a completely different group of pollinators than indicated by the flower features. This means that the pollination syndrome concept does not always guarantee an accurate prediction of the principal pollinator. This may be partly related to the evolution, e.g., when a pollinator changes rapidly due to attraction to a newly derived floral feature. Alternatively, it may be related to poor interpretation, when not all morphological features of flowers are taken into account in the analysis [82,83,84].

### 3.3. Evolution of Floral Characters: Synapomorphies vs. Homoplasies

The general topology of the ITS phylogenetic tree obtained in this study is, in general, congruent with the phylogenetic reconstructions of the *Pinguicula* genus based on ITS sequences from previous papers [51,85]. We recovered two major clades of Central American and Mexican species: the *P. emarginata*–*P. moctezumae* clade and *P. agnata–P. gigantea* clade, as well as taxon *P. albida*, corresponding to clades VII, VIII, and IX, respectively, according to Shimai et al. [51]. We included, for the first time, *P. martinezii* to the phylogenetic reconstruction. Analysis showed that *P. martinezii* belongs to the *P. agnata–P. gigantea* clade and constitutes a sister group to the *P. agnata* and *P. ibarrae* subclade. This result supports the previous classification [57,58], in which *P. martinezii* was placed in the *Agnata* section. The position of *P. esseriana* was unresolved in our analysis. Shimai et al. [51,85], in previous analyses based on ITS sequences, placed *P. esseriana* in clade III/VIII (which corresponds to the *P. agnata–P. gigantea* clade in this work) with 52% and 57% bootstrap support, respectively. *P. esseriana* has long-spurred and colored flowers with visible yellow blotches at the entrance to the spur, indicating psychophily pollination syndrome. 

The evolution of pollination syndromes showed that myophily/mellitophily are plesiomorphic conditions, while psychophily and ornithophily syndromes are derived for *Pinguicula*. This observation is congruent with our previous results obtained from phylogenetic reconstruction based on analyses of trnK/matK sequences [50]. Examples of such adaptations to a new particular group of pollinators in far-related taxa have also been observed in *Fritillaria* genus, where species pollinated mainly by insects include twice-evolved ornithophily syndrome for *F. recurve*, *F. gentneri* from the *Liliorhiza* subgenus (pollinated by hummingbirds), and *F. imperialis* from the *Petilium* subgenus (pollinated by passerine birds). Interestingly, reversal shifts of a pollinator from ornithophily to myophily were also present [75]. Another excellent example of the diversification of pollination strategies within a single genus is presented by *Disa* genus, in which a monophyletic group of 27 studied taxa revealed multiple independent radial evolutions of various pollination syndromes [73].

In his monography, Casper [49], presented a classification of *Pinguicula* genus based on flower morphology, focusing mainly on the color and shape of the corolla. He recognized and divided 46 *Pinguicula* species into three subgenera: *Isoloba* Barnhart, *Pinguicula*, and *Temnoceras* Barnhart. More recently, phylogenetical analysis performed by Shimai et al. [51], including a great majority of currently known *Pinguicula* species (approximately 80% of all species), revealed that previous classifications based on flower morphology are incongruent with phylogenetic reconstructions and very often show the artificial affinity between taxa within a genus. Fleischmann and Roccia [57] and Fleischmann [58] also proposed a new infrageneric classification of *Pinguicula* genus. The authors questioned the “traditional” morphology-based classification of *Pinguicula* genus and agreed that geographical distributions and growth types of *Pinguicula* species basically align with the phylogeny of this genus, in contrast to floral features, which are the consequence of convergence or parallel evolution, as a result of adaptation to a certain group of pollinators. Indeed, in our previous paper, the presence of “feeding” was the result of adaptation and more related to pollination syndrome than phylogeny [50]. In our study, character tracing analysis indicated that some of the floral characters turned out to be synapomorphies, supporting the monophyly of particular clades. Within the *P. agnata*–*P. gigantea* clade, species share floral traits such as the presence of non-glandular trichomes in the middle part and on the edges of corolla petals, as well as the presence of non-glandular trichomes with elongated cylinder, concave-shaped cells. In the case of the *P. emarginata–P. moctezumae* clade, species share features such as the presence of short-stalked capitate glandular trichomes at the base of corolla petals of the upper lip and the presence of apical to obtuse pointed slender non-glandular trichomes. Further character tracing, based on the generated phylogenetic tree, indicated that some of the floral characteristics can manifest as either homoplasies or synapomorphies depending on the specific group or clade under consideration. For instance, the elongated tube occurs as a synapomorphy in the *P. agnata*–*P. gigantea* clade, as well as *P. albida*; however, this feature was also derived independently in *P. hemiepiphytica*, probably as a consequence of convergence. The presence of long-stalked capitate glandular trichomes at the base of corolla petals was derived in the *P. emarginata–P. moctezumae* clade; however, *P. emarginata* lost this feature in reversal to a seemingly ancestral trait. Presence of subclavate non-glandular trichomes can be a synapomorphy to the *P. agnata*–*P. gigantea* clade; however, in the case of *P. esseriana* and *P. moctezumae*, it was revealed to be a homoplastic, possibly resulting from convergence. Similar non-glandular trichomes on the corolla petals of the lower lip occurred in *P. agnata*, *P. albida*, *P. esseriana*, *P. gigantea*, *P. ibarrae*, and *P. martinezii*, as well as appeared in *P. mesophytica,* possibly the consequence of convergence. Morphological features such as non-glandular trichomes with a smooth cuticle surface and few celled branches appeared two (in *P. mesophytica* and *P. agnata*) and three (in *P. hemiepiphytica*, *P. moctezumae*, and *P. martinezii*) times, respectively, in far-related species, possible as a result of parallel evolution. Thus, most of the flower features of *Pinguicula* appear to be associated more with plant adaptation to pollinators and less with phylogeny, reflecting affinity between taxa.

In the Lentibulariaceae family, in addition to *Pinguicula,* two other genera are present: *Genlisea* and *Utricularia.* While the *Genlisea* genus is poorly understood in terms of pollinators [45,86], more is known about pollinators of the *Utricularia* genus. It seems that the situation of *Utricularia* may resemble that of *Pinguicula*, because a wide range of flower-visiting insects have been observed among *Utricularia* species, including bees, butterflies, moths, hawk moths, dipterans, and birds [47,87,88,89]. Hopefully, similar studies related to mapping and tracking of pollination strategies onto phylogenetic reconstruction will be conducted for *Utricularia* and *Genlisea* in the future.

## 4. Materials and Methods

### 4.1. Plant Material

For our study, 12 different species of *Pinguicula* were sampled: *Pinguicula moctezumae* Zamudio & R.Z. Ortega, *P. esseriana* B. Kirchn, *P. moranensis* Kunth, *P. emarginata* Zamudio & Rzed, *P. rectifolia* Speta & F. Fuchs, *P. mesophytica* Zamudio, *P. hemiepiphytica* Zamudio & Rzed, *P. agnata* Casper, *P. albida* Wright ex Griseb, *P. ibarrae* Zamudio, *P. martinezii* Zamudio, and *P. gigantea* Luhrs. The plants we used were cultivated in the Botanical Garden of the Jagiellonian University in Cracow.

### 4.2. Scanning Electron Microscopy

For micromorphological analysis, a minimum of five flowers from 3–5 plant individuals per tested species were collected and examined by scanning electron microscopy. Fresh plant material was fixed in a mixture of 2.5% or 5% glutaraldehyde (Sigma-Aldrich, St. Louis, MO, USA) with 2.5% formaldehyde (Sigma-Aldrich, St. Louis, MO, USA) in a 0.05 M cacodylate buffer (Sigma-Aldrich, St. Louis, MO, USA; pH 7.2) overnight or for several days, washed three times in a 0.1 M sodium cacodylate buffer, and subsequently dehydrated at a critical point using CO_2_. It was then sputter-coated with gold and examined at an accelerating voltage of 20 kV using a Hitachi S-4700 scanning electron microscope, housed at the Institute of Geological Sciences, Jagiellonian University in Cracow, Poland.

### 4.3. Trichome Density Assessment

For trichome density estimation, five flowers from five plant individuals per tested species were collected and examined. Flowers were cut using a razor blade and fixed in 70% ethanol. Images of particular areas of the flowers, on which glandular and non-glandular trichomes occurred, were consistently captured at 20× magnification using the Eclipse E400 light microscope. Each morphological type of glandular and non-glandular trichomes was counted in an area of 1 mm^2^ on various flower parts: corolla petals, tube, and spur.

### 4.4. Phylogenetic Analyses

The phylogenetic analyses of the ITS region (rDNA) were performed using DNA sequences available in the NCBI nucleotide database: *Pinguicula agnata* (AB199752.1, DQ441602.1 and MG310267.1), *P. albida* (AB212095.1 and MG310268.1), *P. emarginata* (AB199759.1 and MG310275.1), *P. esseriana* (AB199760.1), *P. gigantea* (AB199761.1), *P. hemiepiphytica* (AB199764.1), *P. ibarrae* (AB251603.1), *P. martinezii* (MG310278.1), *P. mesophytica* (AB251604.1), *P. moctezumae* (AB199772.1), *P. moranensis* (AB199773.1), and *P. rectifolia* (AB199780.1). The species *Utricularia neottioides* (MG027759.1), *U. reniformis* (MG027778.1), and *U. volubilis* (MG027738.1) were used as the outgroups. The DNA sequences were aligned in MAFFT v. 7 (https://mafft.cbrc.jp/alignment/server/; accessed on 18 April 2022) [90] with default parameters. The best evolutionary model (best-fit), GTR + I + G, was found using MrModeltest v. 2.4 (Nylander, 2004) [91] according to parameter estimation based on the Akaike information criterion (AIC; Akaike, 1973; Burnham and Anderson, 2004) [92,93]. The Bayesian inference was accomplished with MrBayes v. 3.2.6 (Stockholm, Sweden) (Ronquist et al., 2012) [94]. Specifically, Markov chain Monte Carlo (MCMC) simulations were run for 1,000,000 generations, and trees were sampled every 100 generations until an average standard deviation of split frequencies <0.01 was achieved. The first 25% of trees from all runs were discarded as burn-in. The tree was drawn with FigTree v. 1.4.4 (http://tree.bio.ed.ac.uk/software/figtree/; accessed on 25 April 2022). The character tracing was accomplished on the basis of parsimony and ACCTRAN optimization [95].

## 5. Conclusions

Our morphological study indicated that the flower morphology of species with psychophily and ornithophily syndromes is similar, in contrast to the morphology of species with myophily/mellitophily syndrome, most probably as a result of adaption to a principal pollinator. The character tracing analysis indicated that most micromorphological floral traits are potentially related to pollination syndromes, whereas only a small number of characteristics are shared among all species of *Pinguicula*. Future studies should focus on field research to better understand pollination ecology and determine which visitors are effective pollinators of Central American and Mexican *Pinguicula* species.

## Figures and Tables

**Table 1:** Floral morphological traits used in character tracing analysis.

**General Morphology of the Flower**
1. Elongated (apical pointed part) spur
2. Shortened (apical rounded part) spur
3. Short tube
4. Elongated tube
5. Occurrence of contrast color signal at the entrance to the tube
**Distribution of trichomes on corolla and papillae inside of the flower**
6. Glandular trichomes on middle part and edges of corolla petals
7. Non-glandular trichomes on middle part and edges of corolla petals
8. Short-stalked capitate glandular trichomes at the base of corolla petals of upper lip (at the entrance to the tube)
9. Short-stalked capitate glandular trichomes at the base of corolla petals of lower lip (at the entrance to the tube)
10. Long-stalked capitate glandular trichomes at the base of corolla petals (around the entrance to the tube)
11. Non-glandular trichomes on the corolla petals of lower lip (before the entrance to the tube)
12. Papillae at the entrance to the spur
13. Papillae in spur
**Non-glandular trichome morphological types**
14. Apical to obtuse pointed slender non-glandular trichomes
15. Subclavate non-glandular trichomes
16. Clavate non-glandular trichomes
17. Non-glandular trichomes, with elongated cylinder concave-shaped cells
18. Non-glandular trichomes with compact rounded cells
19. Non-glandular trichomes, with rounded apical “head” cell
20. Moniliform non-glandular trichomes
**Non-glandular trichomes features**
21. Non-glandular trichomes with cuticle striations
22. Non-glandular trichomes with (almost) smooth cuticle surface
23. Non-glandular trichomes with outgrowths
24. Non-glandular trichomes with few celled branches
